# Application of response surface methodology for optimization of medium components for the production of secondary metabolites by *Streptomyces diastatochromogenes* KX852460

**DOI:** 10.1186/s13568-017-0388-z

**Published:** 2017-05-15

**Authors:** Taswar Ahsan, Jianguang Chen, Yuanhua Wu, Muhammad Irfan

**Affiliations:** 1 Department of Plant Pathology, Plant Protection College, Shenyang Agricultural University, Shenyang, People’s Republic of China; 2 Department of Biotechnology, University of Sargodha, Sargodha, Pakistan

**Keywords:** *Streptomyces diastatochromogenes*, Bioactive compounds, RSM, Fermentation

## Abstract

A bioactive strain *Streptomyces diastatochromogenes* KX852460 was selected for the production of secondary metabolites to control the target spot disease on tobacco leaves, caused by the *Rhizoctonia solani* AG-3. Peanut meal, soluble starch, NaCl, yeast extract, and ammonium sulphate were identified the best ingredient for high antifungal activity of *S. diastatochromogenes* KX852460 against the *R. solani* AG-3. For the improved production of secondary metabolites, central composite design of response surface methodology was applied in submerged fermentation. The best concentrations of ingredients were peanut meal 4.88%, soluble starch 4.40%, NaCl 0.52%, yeast extract 0.47%, and ammonium sulphate 0.0360%. Study of metabolism changes in the submerged fermentation process was analyzed. Level of the reducing sugar increased, as the total sugar consumed. Amino nitrogen and total sugar decrease tendency, which indicated the growth of bacteria in submerged fermentation batch. Production of secondary and other metabolites influenced the pH of the fermentation batch.

## Introduction

*Rhizoctonia solani* is the major soil borne pathogen and the causal agent of several kinds of diseases in plants throughout the world, and have destructive impacts on the production of tobacco. In the Liaoning province of China in 2006 target spot disease on tobacco leaves was investigated and analyzed that *R. solani* AG-3 was the causal agent of this disease (Wu et al. 2012). Biological control is most potent practice against the *R. solani*, even though environmental stresses impact on efficacies (dos Reis Almeida et al. 2007). In agriculture for the disease management of crops, production of ecofriendly agronomic antibiotic from nature is a growing task. Several actinomycetes species are the source of antibiotic (Clardy et al. 2006). In pharmaceutical products *Streptomyces* have a big part, and similarly agricultural antibiotics are produce by them (Demain and Sanchez 2009; El-Mehalawy et al. 2005).

Medium composition and fermentation conditions may cause effects on the production of secondary metabolites from the *Streptomyces*. In the yield of secondary metabolites these parameters have a key role (Al-Hulu 2013). Minor variations in the composition of fermentation media, can be influenced on the yield and as well as on the metabolic profile of the microbe (Scherlach and Hertweck 2009). By fermentation production of enzymes and growth of microbes, directly related to the medium composition and conditions. Optimization of these parameters became a significant process. To optimize these parameters for the fermentation, several statics approaches are widely utilized (Kumar et al. 2016). Optimized nutrient parameters not only cause to boost 20% of the antifungal efficacies, however also understand its composition (Gao et al. 2016).

Response surface methodology is very beneficial tool to optimize numerous parameters of trails, to find relativeness among the factors, to find the best combination of parameters and prediction of responses. For the optimization of microbial products this method extensively used (Grahovac et al. 2014; Kong et al. 2014). For the optimization of important fermentation parameters several types of designs are accessible, in optimization process central composite design (CCD) is one of the most useful designs (Nouby et al. 2009). In current study an effort has been made to produce antibiotic by *Streptomyces diastatochromogenes* KX852460, using the central composite design of response surface methodology to improve the antifungal activity by optimizing the nutrient components in submerged fermentation.

## Materials and methods

### Microorganism

*Streptomyces* strain TA1123 was isolated and identified as *Streptomyces diastatochromogenes* (GenBank Accession Number KX852460) on the base of morphological, biochemical and 16S ribosomal RNA gene sequence (Ahsan et al. 2017a) and submitted to Chinese general microbial collection center (CGMCC4.7384) (Ahsan et al. 2017b). *R. solani* AG-3 (Gene bank accession number of KX852461, Chinese general microbial collection center CGMCC3.18223) was isolated from infected tobacco leaves and identified through molecular techniques (Ahsan et al. 2017b).

### Inoculum development

Inoculum was prepared as described in our earlier reports (Ahsan et al. 2017b). Briefly, after the development of spores of *Streptomyces* strain KX852460 on Gause’s synthetic medium agar plates, two spore cakes (5 mm) were used to inoculate the fermentation medium for the production of secondary metabolites.

### Mode of fermentation

Submerged fermentation by *S. diastatochromogenes* KX852460 was carried out in 250 ml Erlenmeyer flask containing 55 ml of medium. The medium comprised of (g/l) soluble starch 47.0, Yeast extract 3.0, peanut meal 22.0, (NH_4_)_2_ SO_4_ 2.7, NaCl 2.7, CaCo_3_, pH 6.8 was sterilized at 121 °C, 15 lbs for 15 min. After sterilization, the medium was allowed to cool at room temperature and inoculated with 7% suspension of *S*. *diastatochromogenes* and incubated at 30 °C with shaking speed of 165 rpm for 96 h. After the completion of fermentation, the culture filtrate was assessed for antifungal activity (Ahsan et al. 2017a).

### Selection of the most significant fermentation parameters

Peanut meal and soluble starch were used as carbon and nitrogen sources, yeast added as additional nutrient, while others were kept at original concentrations. Select the most significant parameters by using the single variable procedure. Other microelements were including NaCl (0, 0.2, 0.4, 0.6, 0.8, 1, 1.2, and 1.4%), ZnSO_4_ (0, 001, 0.01 and 0.1%), FeSO_4_ (0, 0.0005, 0.001, 0.0015 and 0.002%), (NH_4_)_2_ SO_4_ (0, 0.2, 0.4, 0.6, 0.8, 1, 1.2 and 1.4%), CuSO_4_ (0, 0.001, 0.01 and 0.1%), CaCO_3_ (0, 0.3, 0.6, 0.9, and 1.2%), MgSO_4_∙7 H_2_O (0, 0.001, 0.01 and 0.1%), MnCl_2_ (0, 0.01, 0.02, 0.03 and 0.04%), ZnSO_4_ (0, 0.001, 0.01 and 0.1%) and KH_2_PO_4_ (0, 0.04, 0.08, 0.12 and 0.16), were added each nutrient to the medium, and antifungal activity was determined by oxford cup method, after 96 h of inoculation under shaking at 165 rpm at 30 °C.

### Experimental design for optimization of nutrient medium

In current study a central composite design (CCD) of five-factor-three-level was used, demanding 32 trials. The fractional factorial design comprises of nine factorial points, fourteen center points, and nine axial points with five parameters. The parameters and their levels used for the optimization of fermentation broth for the production of secondary metabolites was: X_1_: peanut meal (0–12 ml%), X_2_: soluble starch (0–12%), X_3_: NaCl (0–1.2%), X_4_: yeast (0–1.2%), and X_5_: ammonium sulfate (0.00–0.12%) (Table 1). Contour plots were generated to demonstrate the key and interactive impact between the independent variables and dependent variables. On the base of the ridge maxima analysis and canonical analysis, the optimal mishmash of the factors can be demonstrated, by employing the optimization function of the MINITAB 14 software. In the software response optimizer tool determined the optimal value for maximum antimicrobial activity.

**Table 1:** Experiment design and results of optimization of nutrient medium for the production of secondary metabolites from *Streptomyces* 1123 by the central composite design

Run no.	X_1_	X_2_	X_3_	X_4_	X_5_	Inhibition zone (mm)		Residual value
						Observed	Predicted	
1	4	4	0.4	0.4	0.04	30.5	32.0341	0.80684
2	8	8	0.8	0	0	18.9	20.1345	1.52224
3	4	12	0.4	0.4	0.04	20	21.3111	1.56492
4	4	−4	0.4	0.4	0.04	22	19.2044	1.56492
5	0	0	0	0	0.08	13.8	16.1961	1.52224
6	12	4	0.4	0.4	0.04	23	21.4044	1.56492
7	8	8	0	0.8	0	23	21.0378	1.52224
8	0	8	0.8	0	0.08	20.2	19.4878	1.52224
9	4	4	1.2	0.4	0.04	21	19.4811	1.56492
10	0	8	0.8	0.8	0	18.5	16.8995	1.52224
11	8	8	0	0	0.08	14	13.8661	1.52224
12	4	4	0.4	0.4	0.04	33.5	32.0341	0.80684
13	8	0	0	0.8	0.08	11	10.9545	1.52224
14	4	4	0.4	0.4	0.12	24.3	22.2777	1.56492
15	0	0	0.8	0.8	0.08	22.1	21.9161	1.52224
16	0	8	0	0	0	4.5	4.9795	1.52224
17	0	0	0	0.8	0	12	12.4078	1.52224
18	4	4	0.4	0.4	0.04	33.67	32.0341	0.80684
19	0	8	0	0.8	0.08	5.34	5.7511	1.52224
20	4	4	0.4	0.4	0.04	30.35	32.0341	0.80684
21	4	4	0.4	0.4	0.04	32.7	32.0341	0.80684
22	4	4	0.4	0.4	−0.04	18.5	19.0377	1.56492
23	8	0	0	0	0	2.0	1.8628	1.52224
24	−4	4	0.4	0.4	0.04	17.1	17.2111	1.56492
25	0	0	0.8	0	0	3.0	3.0645	1.52224
26	8	0	0.8	0.8	0	20.3	22.2228	1.52224
27	4	4	0.4	1.2	0.04	15	15.6944	1.56492
28	4	4	−0.4	0.4	0.04	8.43	8.4644	1.56492
29	4	4	0.4	0.4	0.04	30	32.0341	0.80684
30	8	8	0.8	0.8	0.08	10	11.1461	1.52224
31	8	0	0.8	0	0.08	13.6	16.2511	1.52224
32	4	4	0.4	−0.4	0.04	11.25	9.0711	1.56492

Coded values; *X*_*1*_ peanut meal, *X*_*2*_ soluble starch, *X*_*3*_ NaCl, *X*_*4*_ yeast, *X*_*5*_ ammonium sulphate

### Metabolic profile in the fermentation batch

Using the dinitrosalicylic acid, reducing sugar in the fermentation batch was measured (Miller 1959) and total sugars were measured by Phenol–sulphuric acid method (Dubois et al. 1956). Amino nitrogen was determined, using ninhydrin reagent (Vu and Le 2010). pH values of fermentation batch was determined at different interval of time using pH meter. Dry cell weight analysis was done by the method of (Wen et al. 2015).

### Statistics analysis

For the fitted experimental results of RSM, regression method of response surface was used. Coded values represented the variables according to the equation:

1 $${\text{Xi}} = \left( {{\text{Xi}} - {\dot{\text{X}}\text{i}}} \right)/{\text{Xi}} = 1,{ 2},{ 3} \ldots {\text{K,}}$$

where x_i_ is an independent variable coded value, X_i_ is the independent variable’s real value, X is the independent variable’s real mean, and X_i_ is the step change value. The second-order polynomial model was fitted a response curve fitting the equation;

2 $${\text{Y}} = {\text{b0 }} + \sum {\text{bixi}} + \sum {\text{i}} \sum {\text{j}} \;{\text{bij xi xj }} + \sum {{\text{bii}}\;{\text{xi}}} ,$$

where Y is stand for measurement of response; the intercept term indicated by b0; bi, bij and bii are measures of the effects of variables xi, xixj and xi respectively. The variable xixj represents the first-order interaction between xi and xj (i < j). Analysis of variance (ANOVA) was used for the statistical analysis of the model, that comprising of the Fisher’s F-test, associated probability P(F), determination coefficient R^2^ and correlation coefficient R that measures the goodness of fit regression model. The analysis also included Student’s t-value for the estimated coefficients and associated probabilities, P(t). For each variable, the quadratic models were represented as contour plots (Gao et al. 2016; Ahsan et al. 2017a).

## Results

### Effect of carbon, nitrogen and microelement sources on antifungal activity of Strain KX852460

**Fig. 1:**
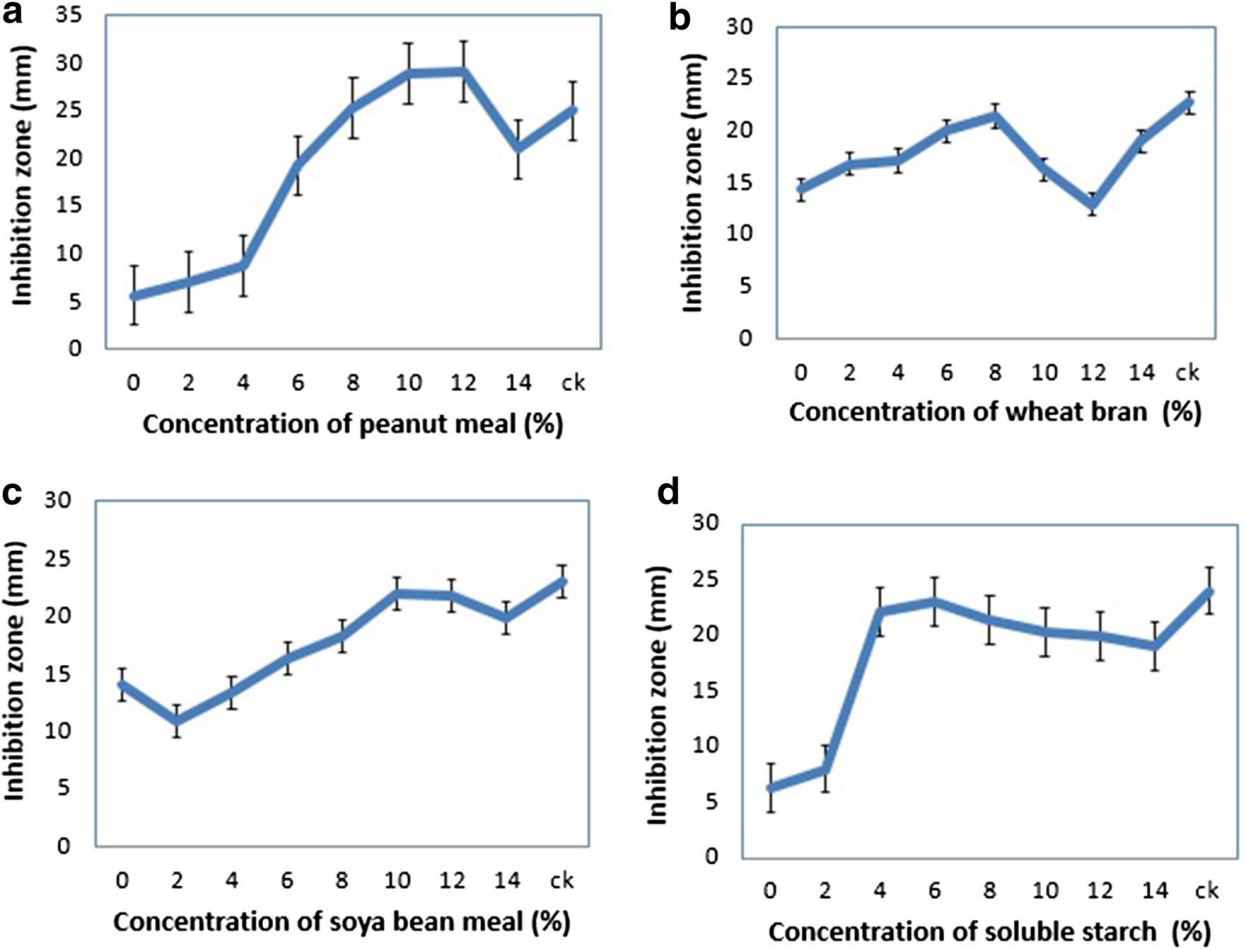
Antifungal effects of nitrogen and carbon sources at different concentrations against the *R. solani AG*-*3*, other nutrients kept at their original concentrations. **a** Peanut meal, **b** wheat bran, **c** soya bean, and **d** soluble starch

Peanut meal, wheat bran, soya bean meal and soluble starch were used as carbon and nitrogen source. Effects of concentrations of these carbon and nitrogen sources in the medium on the antifungal activity were determined. By increasing the concentrations of peanut meal and soluble starch from 0 to 14%, the diameter of inhibition zone increased (Fig. 1a, d), while the antifungal effects of wheat bran and soya bean meal were not significant. Increasing the concentrations of wheat bran and soya bean meal from 0 to 14%, the diameter of inhibition zone was observed not increased as compared to peanut meal and soluble starch (Fig. 1b, c).

**Fig. 2:**
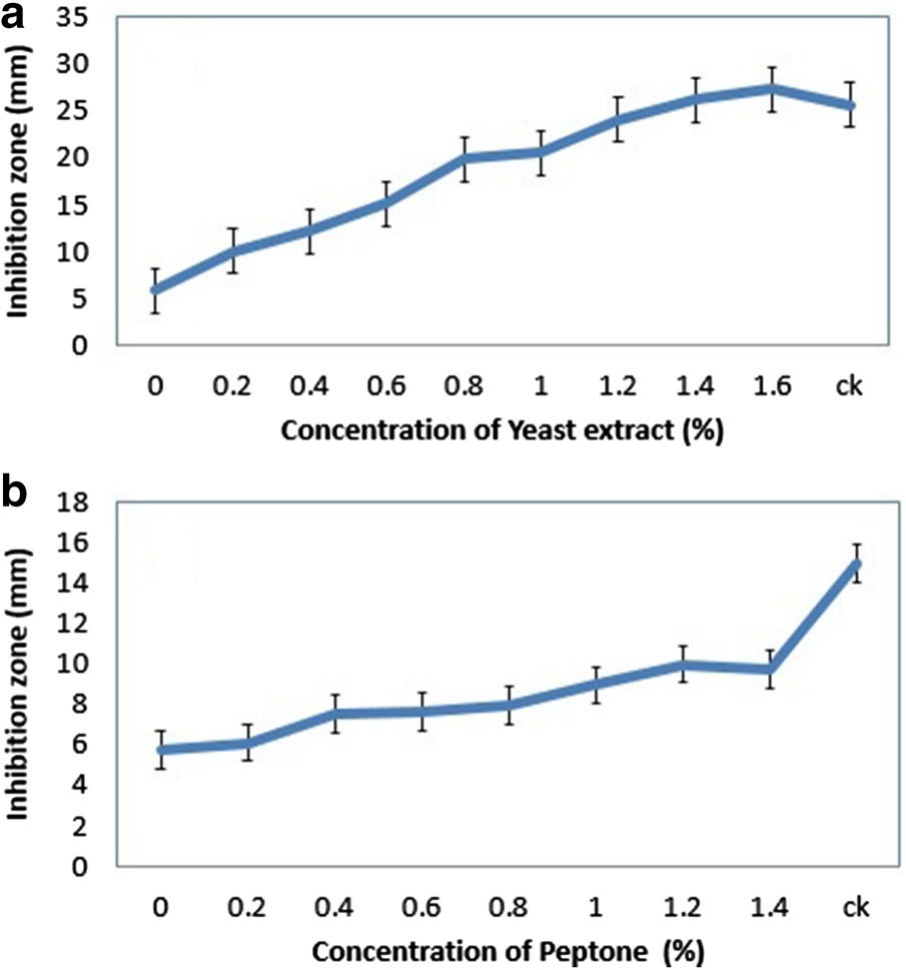
Antifungal effects of supplement compounds at different concentration against the *R. solani AG*-*3*, other nutrients kept at their original concentrations. **a** Yeast extract, and **b** peptone

**Fig. 3:**
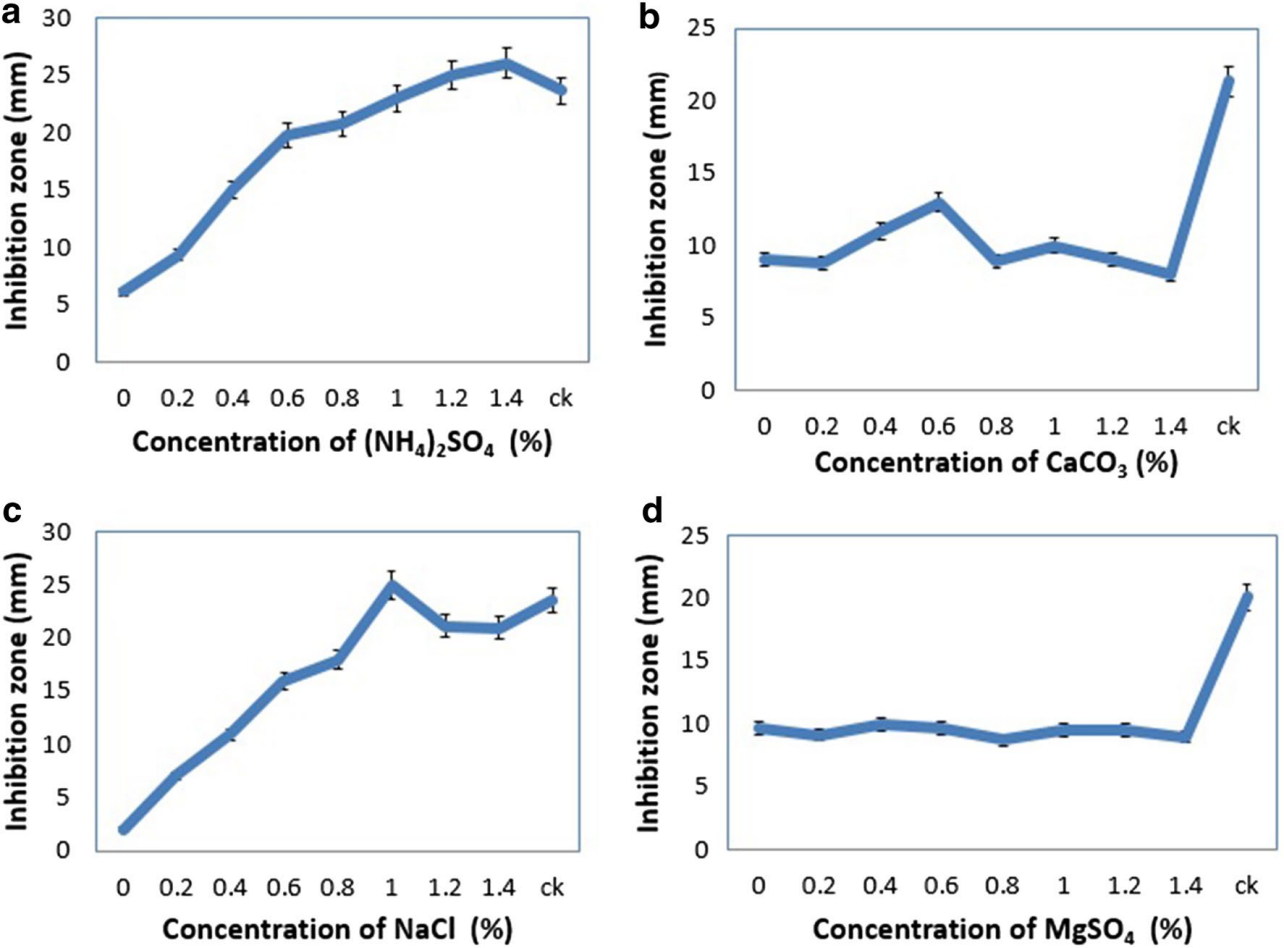
Antifungal effects of microelements against the *R. solani* AG-3 **a** ammonium sulfate, **b** calcium carbonate, **c** NaCl, and **d** magnesium sulfate

Yeast extract and peptone were added in the medium as additional nutrients. Yeast extract had significant antifungal effects, as the concentration increased from 0 to 1.6% (Fig. 2a). While the peptone was investigated with the same concentration and results showed not too much influenced on antifungal activity (Fig. 2b). Four microelements were screened for the maximum production of antibiotic, to the disease management of target spot on tobacco leaf and suppressing the pathogen *R. solani* AG-3. Ammonium sulfate, calcium carbonate, sodium chloride, and magnesium sulphate with same concentration of each ranges from 0 to 1.4%. Ammonium sulfate and sodium chloride had significant effects on the antifungal activity of strain KX852460 (Fig. 3a, c). Calcium carbonate and magnesium sulphate had no significant effects on the antifungal activity against *R. solani* AG-3 by the strain KX852460 (Fig. 3b, d). On the base of results and keeping in view the cost of the experiment, following nutrients i.e. peanut meal, soluble starch, yeast extract, sodium chloride, and ammonium sulphate were screened for the optimization of fermentation medium.

### Optimization of fermentation medium through CCD

The antifungal effects of antibiotic, produced by *S. diastatochromogenes* KX852460 were maximized by optimization of the medium components through central composite design (CCD) of response surface methodology. Thirty-two experiments were conducted in triplicate with different concentrations of Peanut meal, soluble starch, NaCl, yeast extract and ammonium sulfate (Table 1). The response obtained from experiments of central composite design (Table 2) were calculated with second order polynomial multiple regression Eq. 3.

**Table 2:** Analysis of variance of antifungal activity

Source	DF	Adj SS	Adj MS	F-value	*P* value
Model	15	2406.84	160.456	39.21	0.000
Linear	5	296.63	59.327	14.50	0.000
X_1_	1	26.38	26.376	6.45	0.022
X_2_	1	6.66	6.657	1.63	0.220
X_3_	1	182.05	182.050	44.49	0.000
X4	1	65.80	65.803	16.08	0.001
X_5_	1	15.75	15.746	3.85	0.067
Square	5	1607.94	321.588	78.59	0.000
X_1_^2^ X_1_	1	296.93	296.927	72.56	0.000
X_2_^2^X_1_	1	254.25	254.252	62.13	0.000
X_3_^2^ X_3_	1	598.06	598.057	146.15	0.000
X_4_^2^ X_4_	1	707.99	707.990	173.02	0.000
X_5_^2^ X_5_	1	237.27	237.273	57.99	0.000
2-way interaction	5	502.27	100.453	24.55	0.000
X_1_^2^ X_2_	1	28.52	28.516	6.97	0.018
X_1_^2^ X_5_	1	95.26	95.258	23.28	0.000
X_2_^2^ X_4_	1	71.23	71.234	17.41	0.001
X_2_^2^ X_5_	1	92.93	92.930	22.71	000.0
X_4_^2^ X_5_	1	214.33	214.330	52.38	0.000
Error	16	65.47	4.092		
Lack-of-fit	11	51.24	4.658	1.64	0.306
Pure error	5	14.23	2.846		
Total	31	2472.31			

3 $$\begin{aligned} {\text{Antifungal activity }}({\text{Y}}) &= - 2.44 + 2.129{\text{X}}_{1} + 2.400{\text{X}}_{2} + 29.46{\text{X}}_{3} + 43.13{\text{X}}_{4} + 375.2{\text{X}}_{5} \\ &\quad - 0.1988{\text{X}}_{1}^{2} {} - 0.1840{\text{X}}_{2}^{2} - 28.22{\text{X}}_{3}^{2} - 30.71{\text{X}}_{4}^{2} \\ &\quad - 1778{\text{X}}_{5}^{2} + 0.0834{\text{X}}_{1} {\text{X}}_{2} - 15.25{\text{X}}_{1} {\text{X}}_{5} - 1.319{\text{X}}_{2} {\text{X}}_{4} \\ &\quad - 15.06{\text{X}}_{2} {\text{X}}_{5} - 228.7{\text{X}}_{4} {\text{X}}_{5} \end{aligned}$$

where Y is the response (antibiotic activity); and X_1_, X_2_, X_3_, X_4_ and X_5_ are the coded values of the independent factors, viz., Peanut flour, soluble starch, NaCl, yeast and ammonium sulfate respectively.

Analysis of variance (ANOVA) was performed on data collected from experiments as shown in the Table 3. The proposed model for this study was found significant having Fischer test value of 39.21 and *P* value of 0.000. The coefficient of determination R^2^ showed the appropriateness of the adequate model. R^2^ values also determined the trail parameters, their interaction and showed unpredictability in the response. In this study coefficient determination R^2^ = 0.9735 or 97.35% indicated that about 2.65% variations were not determined by the model. The adjusted determination coefficient R^2^ = 0.9487 or 94.87% also showed that the model was highly significant.

**Table 3:** Coded coefficients for antifungal activity

Term	Effect	Coef	SE Coef	T-value	*P*-value	VIF
Constant		32.034	0.807	39.70	0.000	
X_1_	2.097	1.048	0.413	2.54	0.022	1.00
X_2_	1.053	0.527	0.413	1.28	0.220	1.00
X_3_	5.508	2.754	0.413	6.67	0.000	1.00
X_4_	3.312	1.656	0.413	4.01	0.001	1.00
X_5_	1.620	0.810	0.413	1.96	0.067	1.00
X_1_^2^ X_1_	−6.363	−3.182	0.373	−8.52	0.000	1.02
X_2_^2^ X_2_	−5.888	−2.944	0.373	−7.88	0.000	1.02
X_3_^2^ X_3_	−9.031	−4.515	0.373	−12.09	0.000	1.02
X_4_^2^X_4_	−9.826	−4.913	0.373	−13.15	0.000	1.02
X_5_^2^ X_5_	−5.688	−2.844	0.373	−7.61	0.000	1.02
X_1_^2^ X_2_	2.670	1.335	0.506	2.64	0.018	1.00
X_1_^2^ X_5_	−4.880	−2.440	0.506	−4.82	0.000	1.00
X_2_^2^ X_4_	−4.220	−2.110	0.506	−4.17	0.001	1.00
X_2_^2^ X_5_	−4.820	−2.410	0.506	−4.77	0.000	1.00
X_4_^2^ X_5_	−7.320	−3.660	0.506	−7.24	0.000	1.00

**Fig. 4:**
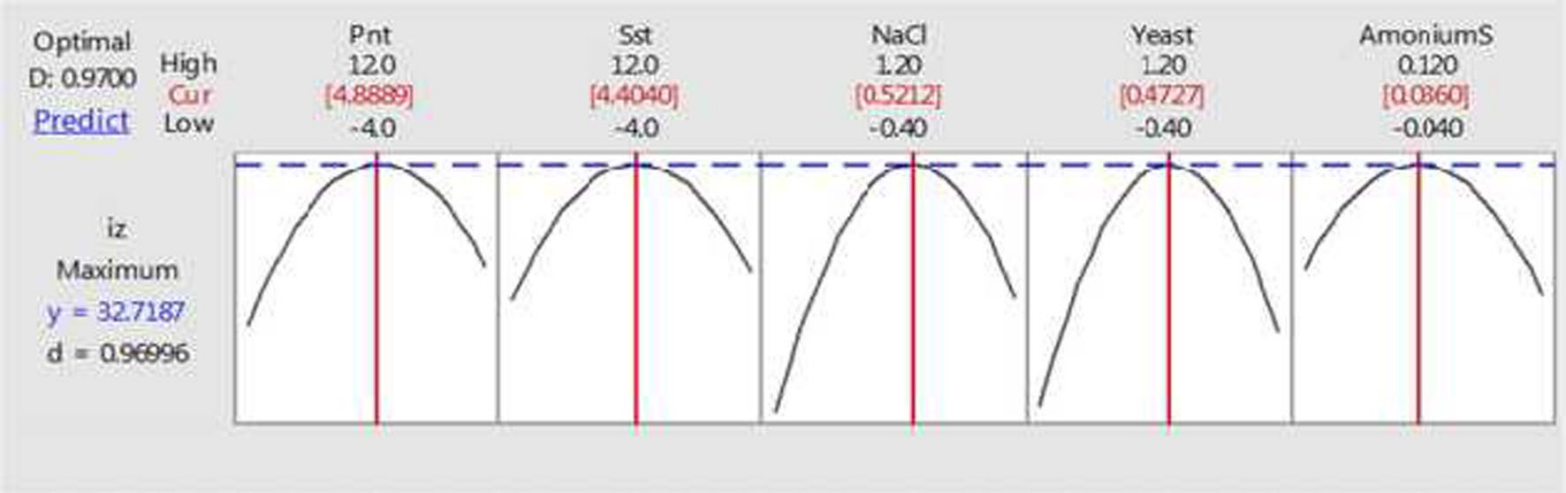
Predicted and actual values of media composition and inhibition zone against the *R. solani* AG-3

All the linear term regression coefficients showed great impact on the antibiotic activity according to the significance of corresponding p-values [pX_1_ = 0.022, pX_2_ = (0.022), pX_3_ = (0.000), pX4 = (0.001), and pX_5_ = (0.067)]. Peanut flour, soluble starch, NaCl, yeast and ammonium sulfate showed their great effect during the activity of the antibiotic. Quadric coefficient of X_11_, X_22_, X_33_, X_44_, and X_55_ were significant and negative effect of these quadrants determined that amplifying the activity of the antibiotic as the parameter’s values enlarged and decreased as the parameter values increased above from certain values. Interactive terms coefficients of X_12_, X_15_, X_24_, and X_45_ were significant. Predicted and actual values of the medium and zone of inhibition against the fungus were given in the (Fig. 4).

**Fig. 5:**
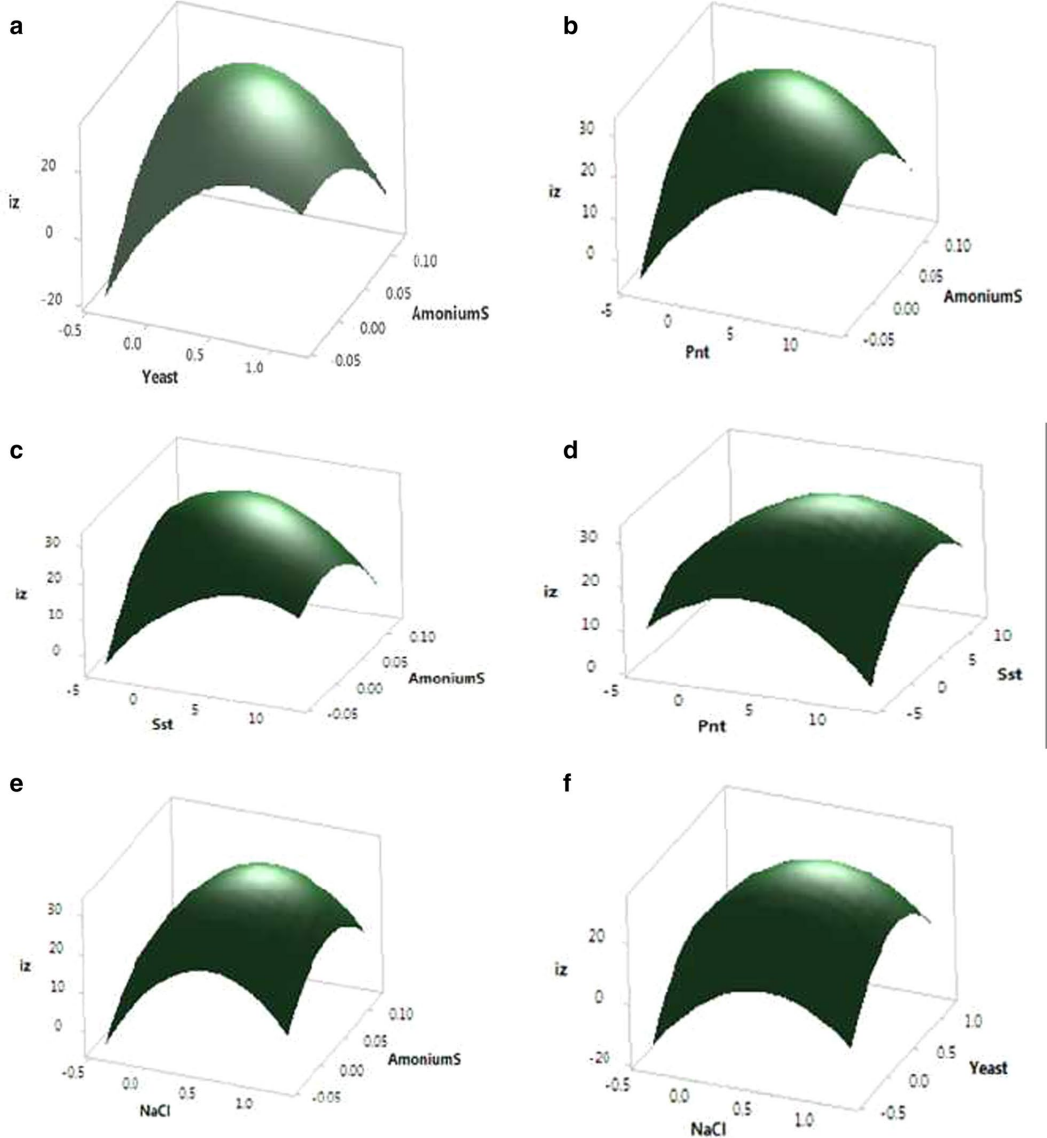
Surface plot of antibiotic activity of *Streptomyces diastatochromogenes* KX852460 diameter of inhibition zone (mm), **a**, the effect of yeast and ammonium sulfate on the inhibition zone, **b** the effect peanut meal and ammonium sulfate on inhibition zone, **c** the effect of soluble starch and ammonium sulfate on inhibition zone, **d** the effect of peanut meal and soluble starch on inhibition zone, **e** the effect of NaCl and ammonium sulfate on inhibition zone, **f** the effect of NaCl and yeast on inhibition zone

**Fig. 6:**
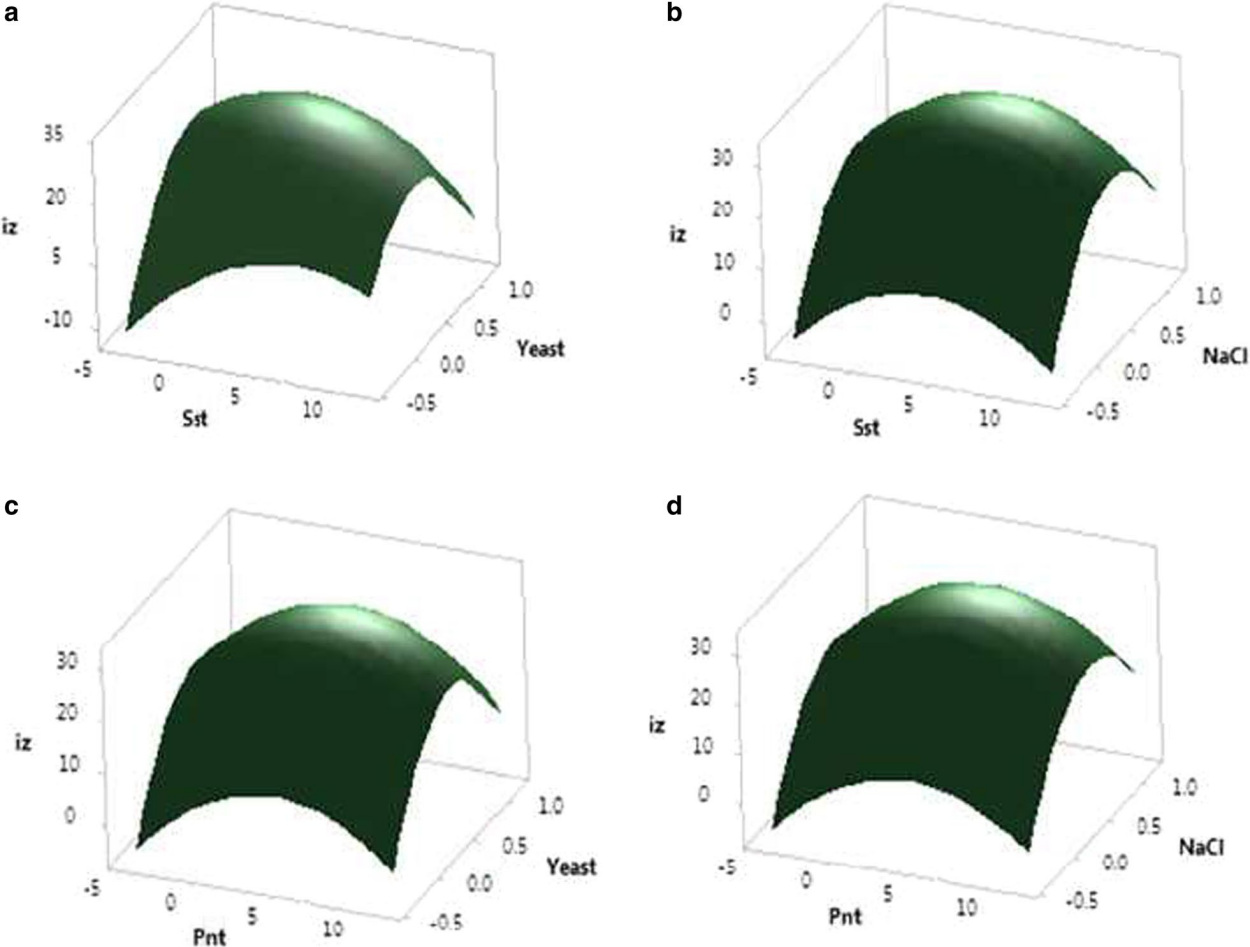
Surface plot of antibiotic activity of *Streptomyces diastatochromogenes* KX852460 diameter of inhibition zone (mm), **a** the effect of soluble starch and yeast on the inhibition zone, **b** soluble starch and NaCl on the inhibition zone, **c** the effect of peanut meal and yeast on inhibition zone, and **d** the effect of peanut meal and NaCl on inhibition zone

Regression models generated surface plots to determine the impact of independent variables and interactive effect of each variable. Signification of variables directly related to the shape of each surface plot that the reciprocal interactions among the independent variables. Figures 5a–f, 6a–d shown the activity for each pair of variables and kept the others constant at their middle values.

### Metabolism of *Streptomyces diastatochromogenes* KX852460 during fermentation in shaking flasks

During the fermentation process evaluation of total sugar, reducing sugar, amino nitrogen, pH and dry mycelium weight was done The total sugar was utilized; at initial stages the concentration of total sugar was high, within the time utilization of total sugar was increased and concentration of total sugar was decreased. At initial stages, the consumption was low, as the total sugar was utilized gradually, concentration of reducing sugar increased, and after 96 h decreased abruptly. Amino nitrogen level was up and down, while pH values was also not stable and remained between 5.5 and 6.6 in the whole process of fermentation. Dry mycelium weight was increased within the hours of fermentation process, after 84 h the weight of dry mycelium was highest, while it became decreased after 120 h of fermentation (Table 4).

**Table 4:** Metabolism study of submerged fermentation batch produced by *Streptomyces diastatochromogenes* KX852460

Parameters	Culture time (h)										
	0	12	24	36	48	60	72	84	96	108	120
Total sugar (mg/ml)	87.3	79.3	75.6	64.2	51.5	39.0	35.0	28.0	20.7	13.1	12.3
Reducing sugar (mg/ml)	0	3.4	4.4	5.3	15.9	18.8	20.0	17.8	15.1	9.0	9.0
Amino nitrogen (mg/ml)	0.55	0.66	0.60	0.61	0.59	0.49	0.37	0.14	0.14	0.10	0.10
pH value	6.6	6.3	6.5	6.5	6.5	6.5	5.9	6.0	5.9	5.6	5.5
Dry mycelium weight (mg/ml)	0	4.4	7.0	15.2	20.0	20.9	21.3	23.2	21.3	20.9	19.5

## Discussion

For the production of antibiotics from the microbes, microelements are very crucial. Several phenomenons including physiologically and synthetically were affected by the concentrations of microelements (Cao and Ma 2002). On the base of antifungal activity against the *R. solani* AG-3, peanut meal and soluble starch were selected for the optimized carbon and nitrogen source for the production of antibiotic in the fermentation medium respectively. Several studies revealed that growth and bioactivity of microbe were greatly affected by the constitution of the substrate medium, such as carbon, nitrogen sources and inorganic microelements (Purama and Goyal 2008; Rao et al. 2007; Song et al. 2007). Current study reveals that yeast extract and peptone had impact on the activity of antibiotic against the *R. solani* AG-3. In the complex fermentation composition yeast extract and peptone had great influence on the production of actinorhodin by *Streptomyces coelicolor* A3 (2) (Elibol 2004). To find optimize parameters response surface methodology is suitable mathematical and statistical tool; it can be helpful to find experimental design for illuminating the relations between the different parameters. Recently RSM was used extensively to optimize fermentation parameters (Bankar and Singhal 2010; Purama and Goyal 2008; Song et al. 2007). Later total sugar, reducing sugar and amino nitrogen level in the fermentation batch decreased, while at the same time growth of bacteria increased. As the total sugar, reducing sugar, and amino nitrogen consumed by the *Streptomyces* strain. The rate of growth of *Streptomyces* strain was declined due to accumulation of secondary metabolites and other metabolites in fermentation batch and pH became decreased. By the utilization of total sugar, the level of reducing sugar decreased, positively effect on pH value and negatively effect on the dry mycelium that led to increase the synthesis of fermentation product (Li et al. 2002). *S. diastatochromogenes* KX852460 had good antifungal effects against the *R. solani* AG-3 to combat the target spot disease on tobacco leaves. Peanut meal, soluble starch, NaCl, yeast extract, and ammonium sulphate were optimized ingredients for the high antifungal activity of secondary metabolites. This product could be good fungicide for the biological disease management of target spot and other *R. solani* related disease.

## Abbreviations

RSM: response surface methodology
CCD: central composite design
CGMCC: Chinese general microbial culture collection
